# Developmental changes in face visual scanning in autism spectrum disorder as assessed by data-based analysis

**DOI:** 10.3389/fpsyg.2015.00989

**Published:** 2015-07-16

**Authors:** Anouck Amestoy, Etienne Guillaud, Manuel P. Bouvard, Jean-René Cazalets

**Affiliations:** ^1^Department of Child and Adolescent Psychiatry, Charles Perrens Hospital, Université de Bordeaux, BordeauxFrance; ^2^CNRS UMR 5287, Institut de Neurosciences Cognitives et Intégratives d’Aquitaine, Université de Bordeaux, BordeauxFrance

**Keywords:** face, eye tracking, spatial statistic, autism, development, face perception

## Abstract

Individuals with autism spectrum disorder (ASD) present reduced visual attention to faces. However, contradictory conclusions have been drawn about the strategies involved in visual face scanning due to the various methodologies implemented in the study of facial screening. Here, we used a data-driven approach to compare children and adults with ASD subjected to the same free viewing task and to address developmental aspects of face scanning, including its temporal patterning, in healthy children, and adults. Four groups (54 subjects) were included in the study: typical adults, typically developing children, and adults and children with ASD. Eye tracking was performed on subjects viewing unfamiliar faces. Fixations were analyzed using a data-driven approach that employed spatial statistics to provide an objective, unbiased definition of the areas of interest. Typical adults expressed a spatial and temporal strategy for visual scanning that differed from the three other groups, involving a sequential fixation of the right eye (RE), left eye (LE), and mouth. Typically developing children, adults and children with autism exhibited similar fixation patterns and they always started by looking at the RE. Children (typical or with ASD) subsequently looked at the LE or the mouth. Based on the present results, the patterns of fixation for static faces that mature from childhood to adulthood in typical subjects are not found in adults with ASD. The atypical patterns found after developmental progression and experience in ASD groups appear to remain blocked in an immature state that cannot be differentiated from typical developmental child patterns of fixation.

## Introduction

Individuals with autism spectrum disorder (ASD) are characterized by social deficits and with faces being the most complex and frequently encountered social visual stimulus, it has been proposed that face scanning processing may be impaired in ASD (Behrmann et al., 2006; for review see Dawson et al., 2005; Golarai et al., 2006; Jemel et al., 2006; Sasson, 2006; Harms et al., 2010; Falck-Ytter and von Hofsten, 2011; Falck-Ytter et al., 2013b). Eye tracking-based experiments have revealed atypical characteristics in visual scanning strategies (Schultz et al., 2000; Klin et al., 2002b; Pelphrey et al., 2002; Dalton et al., 2005; Corden et al., 2008; Hernandez et al., 2009; Nakano et al., 2010; Yi et al., 2013), leading to reduced visual attention to faces and to the development of the excess mouth/diminished eye gaze hypothesis suggesting that the eyes are not meaningful or that they are perceived as threatening (for review see Falck-Ytter and von Hofsten, 2011; Yi et al., 2013). Over the last 10 years, however, it has emerged, that face scanning performance in ASD is a more complex issue than initially assumed.

Understanding how infants, children and adults capture details from their environment is important in trying to unravel how learning and developmental processes take place (Klin et al., 2002a; Boraston and Blakemore, 2007; Falck-Ytter et al., 2013b). Eye tracking techniques allow to efficiently determine how the observer distributes gaze under various monitored experimental conditions and can serve to address a wide range of scientific questions (for review see Boraston and Blakemore, 2007; Falck-Ytter et al., 2013a). Yarbus (1967) first demonstrated that adults display a distinct and ordered pattern of eye movements during face encoding and recognition, with fixations primarily converging on core facial features, i.e., eyes and mouth that form a triangular scanpath. This template routine has been partially replicated in other studies (Groner et al., 1984; Henderson et al., 2005), which leads to the presumption that such a triangular scan trajectory represents a strategy employed universally by individuals as the most efficient way to extract visual information.

Studies using static or dynamic stimuli have established that subjects with ASD spend a lower percentage of time watching core facial features, whereas they view non-core feature areas more frequently (Dalton et al., 2005; Jemel et al., 2006; Spezio et al., 2007a; Corden et al., 2008). In contrast, other studies have failed to find any differences between ASD patients and matched control subjects (Lahaie et al., 2006; Spezio et al., 2007b; Fletcher-Watson et al., 2009). With specific consideration of the mouth region, the results also remain unclear, since the differences between groups were small, particularly when static neutral pictures were used (for review see Klin et al., 1999; Jemel et al., 2006; Rutherford and Towns, 2008; Falck-Ytter and von Hofsten, 2011; Rice et al., 2012; Falck-Ytter et al., 2013b).

Although the findings from various studies may differ according to the type of stimuli used (Boraston and Blakemore, 2007) or to the participant’s age, atypical scanning strategies especially concerning the time spent on the eye region, have been reported very early in development. Infants subsequently diagnosed with ASDs exhibit a decline in eye fixation within the first 2–6 months of life, a pattern not observed in infants who do not develop ASD (Jones and Klin, 2013). In contrast, Chawarska et al. (2012) did not find marked differences between typical infants and infants later diagnosed with ASD in the distribution of their attention to eyes or mouth, although the ASD group exhibited a weaker attention to a social scene and the face compared to objects of the scene. However, these two experimental situations differ in terms of the level of directness of stimulation, with infant-directed speech being used in the Jones and Klin (2013) experiment. Furthermore, the excess mouth/diminished gaze effect seems to be strongly dependent on dynamic aspects of the stimuli (Falkmer et al., 2011) and whether the video’s actor is addressing, or not, the participants (Chawarska et al., 2012, 2013).

Furthermore, even in a ‘typical’ population, the developmental course of face scanning is to date poorly understood. Therefore, one crucial remaining issue relating to the excess mouth/diminished eye gaze hypothesis is understanding the typical developmental evolution of attention to face, and the way attention shifts between the core facial features (Pascalis et al., 2011). The differences found across studies may be related to the age of participants but also, as recently highlighted, to cultural differences. Wheeler et al. (2011) recently reported that 6-months-old infants fixate significantly more on the left eye (LE) and mouth of own-race faces, but more on the nose of other-race faces. Furthermore, the importance of the core features may vary with age. Specific human eye attraction seems to be absent in newborns but emerges from 3 months of age and remains stable thereafter (Dupierrix et al., 2014) suggesting the importance of experience in the core feature scanning strategy and role for face recognition in humans. Along the same line, when they had to recognize face parts independently of the entire face, 13- to 14-years-old children had already reached adult performance levels in their recognition of the eye region, while their mouth recognition ability continued to develop beyond 14 years of age (Liu et al., 2013). Altogether, these studies suggest that the developmental trajectory of face scanning is a more complex issue than initially thought, and is likely to be only understood through the combined contributions of the various experimental approaches.

Part of the contradictory conclusions drawn in the various studies to date may also come from methodological pitfalls. In eye tracking studies, the definition of the regions of interest (ROIs) considered for analysis relies on experimenter subjectivity, since there is no consensual rules to delineate them (e.g., Henderson et al., 2005; Barton et al., 2006; Orban de Xivry et al., 2008). It is only recently that several studies have raised this issue in proposing quantitative measurement of visual scenes (Over et al., 2006) or data-driven approaches that allow making an *a posteriori* definition of visual targets in a scene (Caldara and Miellet, 2011; Falck-Ytter et al., 2013b; Yi et al., 2014).

Altogether, the various methodologies thus far implemented in the study of typical facial screening – i.e., the type of stimuli, type of task, participant age, the ROIs delineated – makes it difficult to actually define the strategies involved in visual face scanning. The aim of this paper was (1), to propose a data-driven approach that allows defining *a posteriori* the spatial locality of fixation clusters based on spatial statistical methods, using a Dirichlet tessellation, to avoid a subjective definition of ROIs by the experimenter; this was conducted by additionally normalizing all images and data to a single space, and (2) to address developmental and pathological aspects of face scanning in healthy children and adults using this data-driven approach and to compare the performances of children and adults with ASDs in the same free viewing task.

## Materials and Methods

### Subjects

Fifty four subjects divided into four groups were included in the study (**Table 1**). The groups consisted of: (1) typical adult (TD-A); (2) typically developing children (TD-C); (3) adults with autism spectrum disorders (ASD-A); (4) children with autism spectrum disorders (ASD-C). Individuals with Asperger’s syndrome or high functioning autism were all recruited from the Bordeaux Autism Resource Centre. They were diagnosed with ASD by two child psychiatrists according to DSM-IV-TR criteria (American Psychiatric Association, 2000), Autism Diagnostic Interview-Revised (ADI-R) and Autism Diagnostic Observation Schedule (ADOS, Module 3 for younger adolescents and Module 4 for older adolescents and adults) criteria. The intelligence quotient (IQ) was evaluated for subjects with ASD only, using the Wechsler Intelligence Scale for Children-Fourth Edition (WISC IV) and the WAIS for the adult sample. No individual subject had a full IQ lower than 85. All subjects had normal or corrected vision, and no history of neurological disorders. Ocular dominance was determined for each subject by using the Dolman’ hole in the card test (Pointer, 2001; Ehrenstein et al., 2005; Rice et al., 2008; Hernandez et al., 2009). The characteristics for all groups are detailed in **Table 1**. Adult subjects gave their written informed consent and parental permission was obtained for each child, and the protocol in accordance with ethical guide-lines was approved by the ethical research committee (Comité de Protection des Personnes Bordeaux A CPP N° 100038-80).

**Table 1:** Subject profiles.

	TD-A	TD-C	ASD-A	ASD-C
N subjects (M/F)	12/1	11/3	12/1	11/3
Mean age, years (SD)	25.4 (3.9)	11.6 (2.2)	23.8 (3.6)	11.3 (2.1)
Verbal intelligence quotient (IQ) (SD) (WISC IV for Children sample et WAIS for adult sample)	NA	NA	107.2 (18)	94.5 (17)
Non-Verbal IQ (SD) (WISC IV for Children sample et WAIS for adult sample)	NA	NA	102.1 (15)	89.8 (11)
Right ocular dominance, N subjects	9 (69%)	10 (71%)	10 (77%)	9 (64%)
Autism Diagnostic Interview-Revised (ADI-R), Social interaction (SD)	NA	NA	16.5 (2.5)	17.1 (5.07)
Autism Diagnostic Observation Schedule (ADOS) Total	NA	NA	7 (4.5)	8.6 (3)
ADOS, Social Interaction (SD)			5 (2.5)	6.2 (2.3)
ADOS, Communication (SD)			2.3 (2.1)	2.6 (1.2)
ADOS, Restricted Repetitive Behavior			0.8 (0.8)	1.1 (1.3)

*Abbreviations as in previous figure legends. There was no significant difference between groups for ocular dominace (Khi-squarred = 1.35, df = 3, *p* = 0.72). F, female; M, Male. Mean values ± SEM*.

### Procedure

**FIGURE 1:**
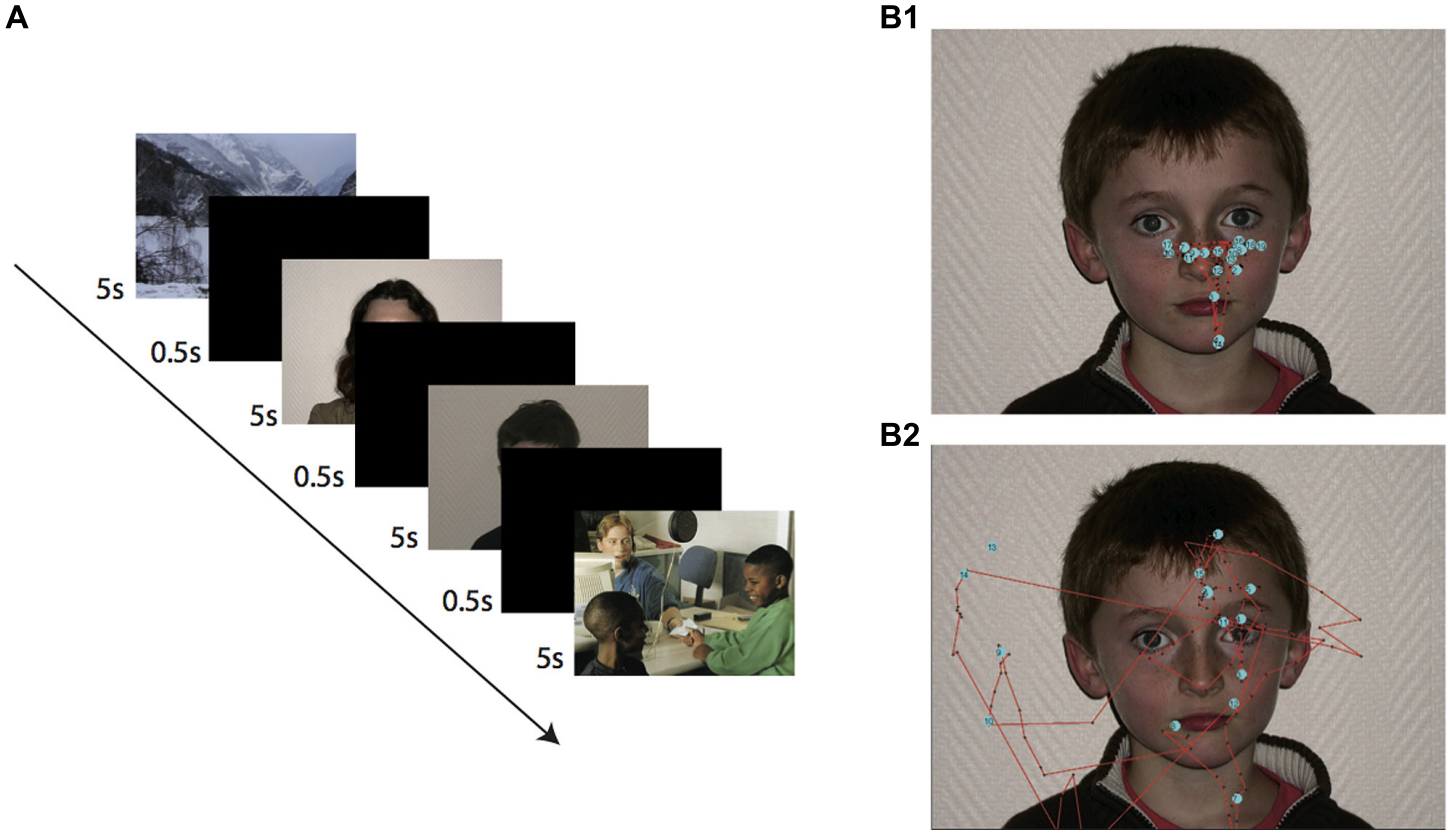
**Experimental task and recording methodology. (A)** Sequence of the different displays presented during the task. The task was sequentially incremented by presentation of up 20 different photographs (here shown only for the four first slides). **(B)** Fixations and visual trajectories for a typical adult subject **(B1)** note the stereotyped triangular pattern of fixation and a child with ASD **(B2)**. Each blue point corresponds to a single fixation.

**Figure 1A** presents schematically the experimental protocol. Each photograph was presented for 5 s and separated with a black screen for 500 ms. A white cross indicated the starting point of fixation between pictures in the central start position of the screen (Althoff and Cohen, 1999). This presentation duration was chosen to avoid overloading the visual pathway (Books et al., 1986; Hernandez et al., 2009).

Two series of 20 color pictures were presented to participants. The presentation of landscape and social scenes also reduced loss of attention and made the task more entertaining. A 2 min pause was made between the presentation of the two series to limit disengagement from the visual stimuli and loss of attention. The two series included 22 neutral unknown faces (11 male children aged between 8 and 16 years, six images of adult males and five of adult females) and 18 images that did not include neutral faces (four pictures of landscapes and 14 of scenes containing people). The order of presentation of the two series was randomized between participants. The pictures were presented on a 15′ monitor viewed from a distance of 60 cm. Photographs of neutral faces positioned centrally in front of a white wall (**Figure 1**) were originally taken of laboratory colleagues, their children, and relatives from a frontal view and chosen from a pool of 50 pictures in which distinguishing marks were absent. Participants were requested to look only at the images and they were unfamiliar with the presented faces.

**FIGURE 2:**
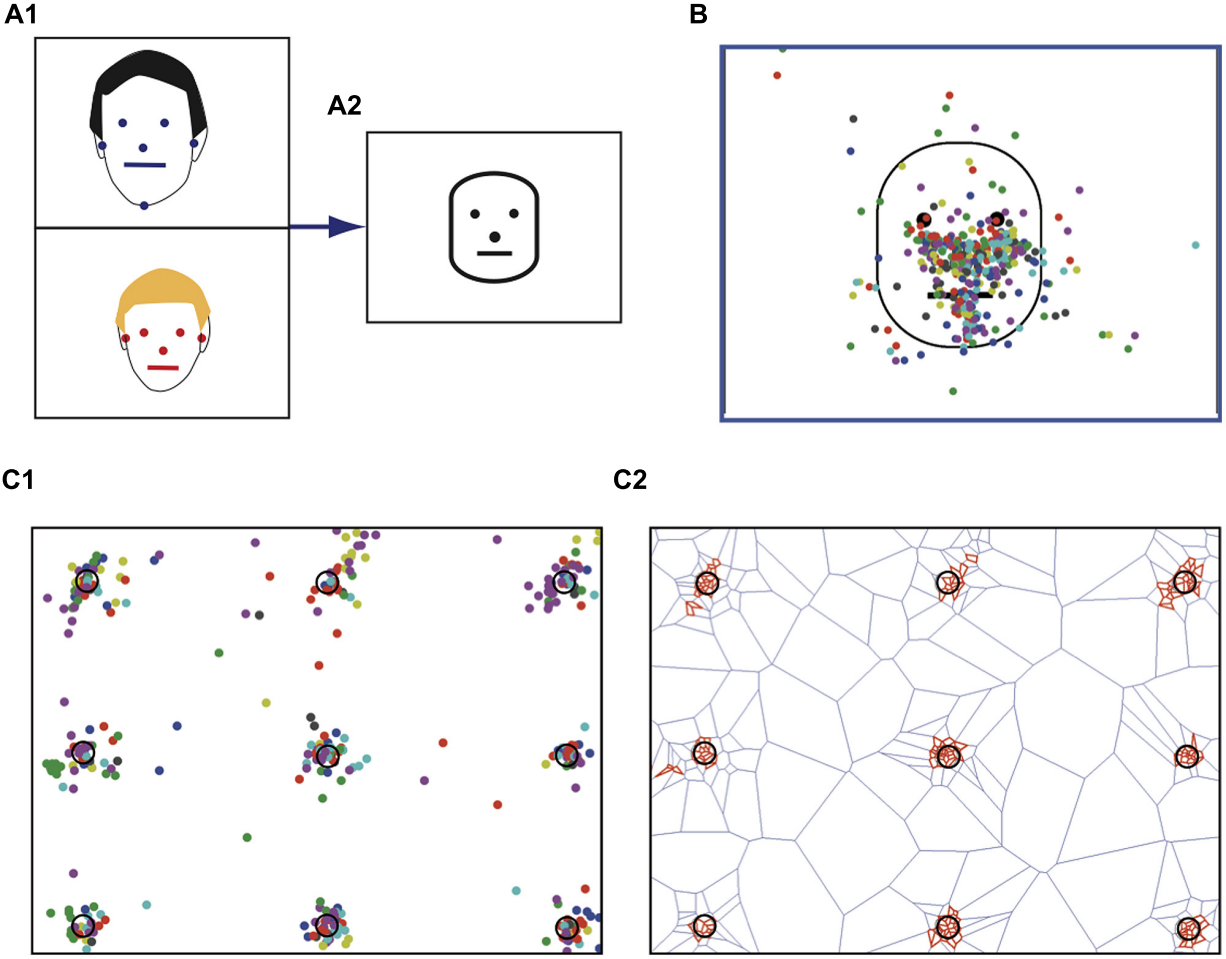
**Spatial normalization of stimulus faces. (A1)** For each face the following anatomical landmarks were identified: the two pupils, tip of the nose, lip commissures, and the ear tragus. **(A2)** Example of a normalized prototypic face. **(B)** Face resulting from the average of 22 stimulus faces. The colored dots (one color per face) represent the fixations performed by a single typical subject for each stimulus face presented. **(C)** Fixations on the calibration map. **(C1)** Each subject had to stare at the various targets. Each color dot corresponds to one subject. **(C2)** Statistically significant clusters of fixation (red tiles) detected using Dirichlet tessellation.

Eye tracking was performed using a remote R6 system (ASL, Bedford, MA, USA) that was operated according to ASL guidelines. A nine point calibration was conducted with each participant at the start of the experiment using the calibration card provided by ASL (**Figure 2C1**). Thereafter, each subject was requested to stare at each point of the calibration card and the associated fixations were recorded (**Figure 2C1**). At the end of the session, we checked that the calibration had not varied by presenting again the 9 point calibratio Arizpe Arizpe Arizpe *n* test card and recording associated fixations. Data were collected with the dedicated ASL software.

### Analysis

Prior to any data processing, we performed a visual inspection of individual raw data traces as those presented in **Figure 1B**, which provides characteristic data examples of the fixation patterns for a typical adult (**Figure 1B1**) and an ASD child. Off-line analysis was subsequently performed using homemade software developed with Matlab (Mathworks, Natick, MA, USA). To obtain comparable data from the various photographs, we performed a spatial normalization of the presented faces using several anatomical landmarks as references (**Figure 2A1**). These included the two pupils, the tip of the nose, the lip commissures and the ear tragus, which together allowed the construction of a prototypic face by homothetic normalization (**Figure 2A2**). We analyzed visual fixation (**Figure 2B**) defined as the point-of-regard when looking at the stationary target. A fixation was calculated as the mean *X* and *Y* eye position coordinates measured over 100 ms during which the eye did not move through a visual angle of more than 1°, according to ASL recommendations.

For all faces presented, the associated recorded fixations were similarly homothetically re-sampled in order to match the prototypic face (**Figure 2B**). The analyzed parameters were: (1) the number of fixations; (2) the total fixation time; (3) the latency of the first fixation within a specific area; (4) fixation duration. Time when fixations were not recorded included blinks, saccades, and time spent looking away from the screen.

### Spatial Statistics

We tested the presence of statistically significant clusters of fixations by using the Dirichlet (or Voronoi) tessellation method, a measure to detect spatial clustering. For a given fixation point *p* in a geometric pattern *X,* a polygon is drawn to create a cell around each point. The cell represents the area in space that is closer to that fixation point than to any other point of *X* (Okabe et al., 1992; Duyckaerts and Godefroy, 2000). Thus, for points in clusters, the closer the points the more the polygons are small. Statistically significant clusters were identified by comparing Dirichlet cell sizes from the actual data to cell areas obtained from surrogate data sets in which point coordinates from the original data set were spatially randomized using the quadrature resample command. Because the logarithm of polygon sizes from surrogate data sets approximated a normal distribution, estimates of the 95% confidence interval (CI) for log polygon sizes from randomized distributions were obtained from 10 surrogate data sets. Points associated with a contiguous Dirichlet polygon obtained from the actual data points whose logarithmically transformed size was smaller than the 95% CI of the surrogate data set polygon sizes were considered constituents of statistically significant clusters. All analyses were performed using MatLab. **Figure 2C** illustrates the procedure, using the calibration map as an example. In this case, adult subjects were requested to look successively at the various spots (numbered 1–9, **Figure 2C1**) on the screen. The Dirichlet based clusterization (**Figure 2C2**), revealed that gaze was mainly centered on the spots, although subjects could occasionally view some other part of the image or in the vicinity of each spot. As indicated above, for each subject two fixation data sets from the calibration map were acquired, at the beginning and at the end of the experiment, in order to validate our experimental measurements.

Statistical analyses were performed using IBM SPSS Statistics software (IBM Corporation, USA). Unless otherwise specified, values are given as mean ± SE of the mean (*M* ± SEM) and were considered to be significantly different at *p* < 0.05. Comparisons among groups and ROI for each variable were performed using non-parametric Kruskal–Wallis *H* test (as described by Laerd statistics, London, UK) as all data set values were not normally distributed. Subsequent *post hoc* analysis were performed using Dunn’s procedure with a Bonferroni correction for multiple comparisons. Adjusted *p*-values and effect size (r) are presented. Occasionally the *p* value was so small that it was expressed as *p* < 0.001. Correlations were made with Pearson’s test.

## Results

### Overall Characteristics of Visual Scanning

We first performed a global analysis of all fixations on the 22 neutral unknown faces, in the four groups. One-way ANOVA analysis revealed that the typical adult group differed significantly from the TD-C and the two ASD groups for all selected variables (**Table 2**). There was no significant difference between groups in terms of fixation duration (*p* = 0.49). Kruskal–Wallis test indicated that there were differences between groups in the number of fixations, *H*(3) = 53, *p* < 0.001, *r* = 0.05. Typical adults performed a higher number of fixations than ASD-A (*p* < 0.001) and ASD-C (*p* < 0.001) but not TD-C (*p* = 0.059) as revealed by *post hoc* analysis. Kruskal–Wallis test also indicated that there were differences between groups in the total time spent at viewing the photographs, *H*(3) = 110, *p* < 0.001, *r* = 0.1. TD-A spent significantly more time at fixating the photographs than the three other groups (*p* < 0.001 for all pairwise comparisons). Kruskal–Wallis test indicated that there were differences between groups in the delay to first fixations, *H*(3) = 62, *p* < 0.001, *r* = 0.06. The delay to the first fixation was significantly lower in typical adults than in other groups (*p* < 0.001 for all pairwise comparisons). The total fixation time, which depended on the individual fixation durations, was positively correlated with the number of fixations, Pearson’s *r*(1089) = 0.52, *p* < 0.001. Therefore, for subsequent analyses, we considered only the time spent in a given area. We also calculated the proportion of time spent viewing a face in comparison with the total time spent viewing a picture (last lines, **Table 2**). The two groups with ASD spent less time scrutinizing faces than typical adults.

**Table 2:** Fixation parameters for all four groups.

	TD-A	TD-C	ASD-A	ASD-C
N subjects	13	14	13	14
Fixation duration, ms (SD)	0.32 (0.00)	0.32 (0.01)	0.3 (0.00)	0.34 (0.02)
Number of fixations	13.8 (0.2)^∗^	12.7 (0.3)	12 (0.2)	11.7 (0.2)
Delay to first fixation, s (SD)	0.05 (0.00)^∗^	0.13 (0.02)	0.1 (0.02)	0.13 (0.01)
Total time spent on photograph, s (SD)	4.8 (0.02)^∗^	4.4 (0.06)	4.4 (0.05)	4.3 (0.05)
% time on face vs image	85%^∗^	78%	80%	73%

Mean values ± SEM. *indicates a significant variation. Abbreviations as in previous figure legends.

### A Data-Driven Approach: Comparison between “*a Priori*” versus “*a Posteriori*” Methodology

**FIGURE 3:**
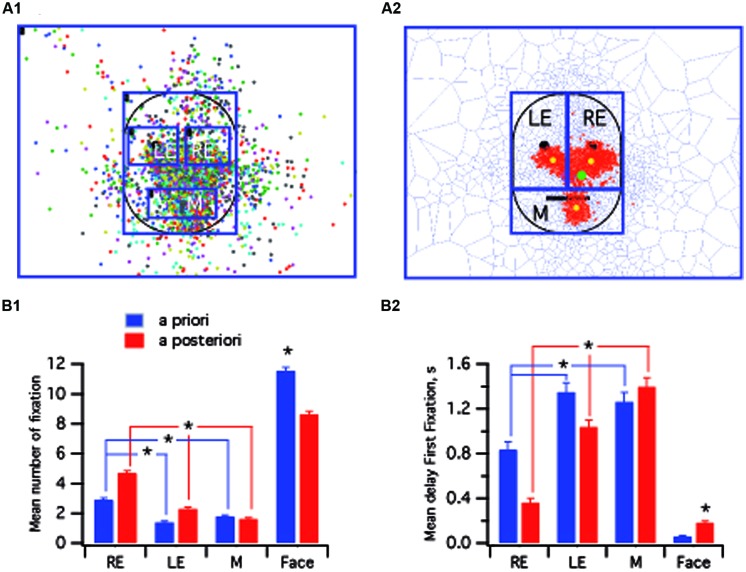
**Comparison between *a priori* and *a posteriori* methods. (A,A1)** Prototypic face with superimposed fixations performed by tested typical adults (*N* = 13 subjects) for all neutral non-familiar faces (*N* = 22 presented faces). Each colored dot corresponds to one subject. The regions of interests (ROIs) were centered on core features (eyes, mouth). **(A2)** From the pattern of superimposed fixations presented in **(A1)**, statistically significant clusters of fixation (red tiles) were detected using Dirichlet tessellation. Large ROIs, not centered on core features (see yellow spots) were circumscribed. **(B)** Bar graphs presenting the mean number of fixations **(B1)** and the mean delay to the first fixation **(B2)** per subject in the four ROIs. M, mouth; LE, left eye; RE, right eye. ^∗^ indicates a significant variation.

One main goal of this study was to test the validity of a data-driven approach to analyzing all four groups. We therefore compared the two methods by using data collected from typical adult subjects presented with 22 non-familiar faces. The overall fixations on the prototypic face (see Materials and Methods) are presented in **Figure 3A1** In the first analytical procedure, termed “*a priori*,” ROIs were defined in accordance with previous studies (Arizpe et al., 2012), that investigated the gaze pattern of face recognition. We defined five ROIs centered on anatomical landmarks: RE, LE, mouth (M), face (F), and out of face (OF), from the observer’s perspective, and the fixations in each ROI were then analyzed. The second analysis procedure, termed “*a posteriori*”, was based on the fixation clusters resulting from the Dirichlet tessellation method (**Figure 3A2**). In this case, the face was divided into three large ROIs that were named RE, LE, and mouth (M), corresponding to the core features (Yarbus, 1967). In this procedure, there was not a direct link between the ROIs and anatomical landmarks that were no longer points of reference. Interestingly, in accounting only for fixations that were encompassed in statistically significant clusters (red tiles **Figure 3A2**), the fixation distribution pattern for each ROI was revealed. The barycenter for each ROI (yellow dots) was clearly located below the eye pupils and the mouth whereas the barycenter for the whole face was located in the right infraorbital zone (green dot). Kruskal–Wallis test indicated that there were differences between zones for both the *a posteriori* [number of fixations: *H*(2) = 276, *p* < 0.001, *r* = 0.33; delay of first fixation : *H*(2) = 240, *p* < 0.001, *r* = 0.33] and the *a priori* methods [number of fixations: *H*(2) = 103, *p* < 0.001, *r* = 0.12; delay of first fixation : *H*(2) = 59, *p* < 0.001, *r* = 0.09]. *Post hoc* analysis indicated that when considering the face, more fixations were taken into account by the *a priori* method (*p* < 0.001; **Figure 3B1**). Indeed, many of the fixations performed by the subjects were not significantly clustered on the three ROIs of interest, i.e., the RE, LE, and mouth. Nevertheless, although a substantial number of non-significant fixations was eliminated by the *a posteriori* analysis overall, it revealed differences that were not apparent with the *a priori* method. With the data-driven approach, the number of fixations was significantly higher on the RE versus the mouth and LE (*p* < 0.001; compare bar graph in **Figure 3B1**). The *a posteriori* analysis also provided more information on the pattern of fixations since in this condition, statistically significant differences were also observed between zones that could not be revealed with the *a priori* method. (**Figure 3B1**) and the fixation delay was longer for the mouth versus the two eyes (*p* < 0.001; **Figure 3B2**). In the subsequent analysis therefore we will only use the *a posteriori* data-driven approach.

### Developmental and Pathological Aspects of Face Scanning: Importance of the Eye Region

**FIGURE 4:**
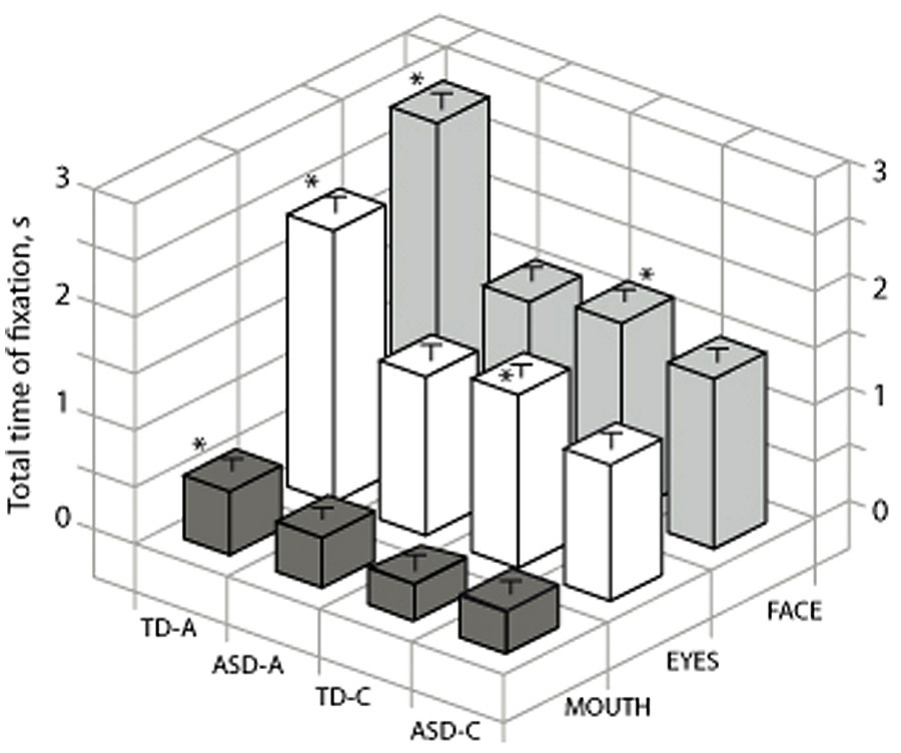
**Comparison for the four groups of total time spent on the eyes and mouth.** TD-A, typical adult; TD-C, typically developing children; ADL/ASD, adults with autism spectrum disorders; ASD-C, children with ASD. Stars on the graph indicate significance between groups for each ROI as for all four groups the time spent in each ROI was significantly different (see text). ^∗^ indicates a significant variation.

Using the *a posteriori* data-driven approach we addressed the role of the eye region (i.e., including both LE and RE) by comparing the time spent on this area compared to the mouth and face (**Figure 4**). Kruskal–Wallis test indicated that for all four groups there were significant differences for the time spent in each ROI [TD-A, *H*(2) = 536, *p* < 0.001, *r* = 0.5; ASD-A, *H*(2) = 344, *p* < 0.001, *r* = 0.2; TD-C, *H*(2) = 296, *p* < 0.001, *r* = 0.33; TD-A, *H*(2) = 234, *p* < 0.001, *r* = 0.26]. Kruskal–Wallis test also indicated that there was significant differences between groups for the time spent on the eye region [*H*(3) = 213, *p* < 0.001, *r* = 0.2] and the mouth region [*H*(2) = 296, *p* < 0.001, *r* = 0.07]. *Post hoc* analysis indicated that typical adults spent more time on the mouth region than the three other groups (TD-A versus TD-C, *p* < 0.001; TD-A versus ASD-A, *p* < 0.001; TD-A versus ASD-C, *p* < 0.001). TD-C also spent significantly more time on the eyes than ASD children (*p* = 0.017). Altogether, these data indicate that individuals with ASD favor focusing on the eye region as typically developing subjects.

Correlation analysis on the whole ASD population (adults and children) was performed to check if social impairment measured by the underscore of social reciprocal interactions from ADI-R and ADOS could be related to the eye tracking data (lower reciprocal social interaction scores on the ADI-R indicate less impairment; higher scores on the ADOS indicate more impairment). We found that the time spent on the eye region was negatively correlated with this ADI-R/RSI [Reciprocal Social Interaction sub scale; Pearson’s *r*(27) = 0.45, *p* < 0.02], indicating that the individuals with ASD who had the lowest score for reciprocal social interaction anomalies looked at the eye region for a longer time. Furthermore, the time spent on the mouth region was correlated with the global score of ADOS [Pearson’s *r*(27) = 0.4, *p* = 0.039], which indicated that ASD individuals with the highest score for social interactions and communication impairment also looked at the mouth region for longer. Other variables, i.e., verbal IQ and age, were not significantly correlated to any eye tracking data.

### Determination of Face Scanning Strategy in Typical versus ASD Subjects

**FIGURE 5:**
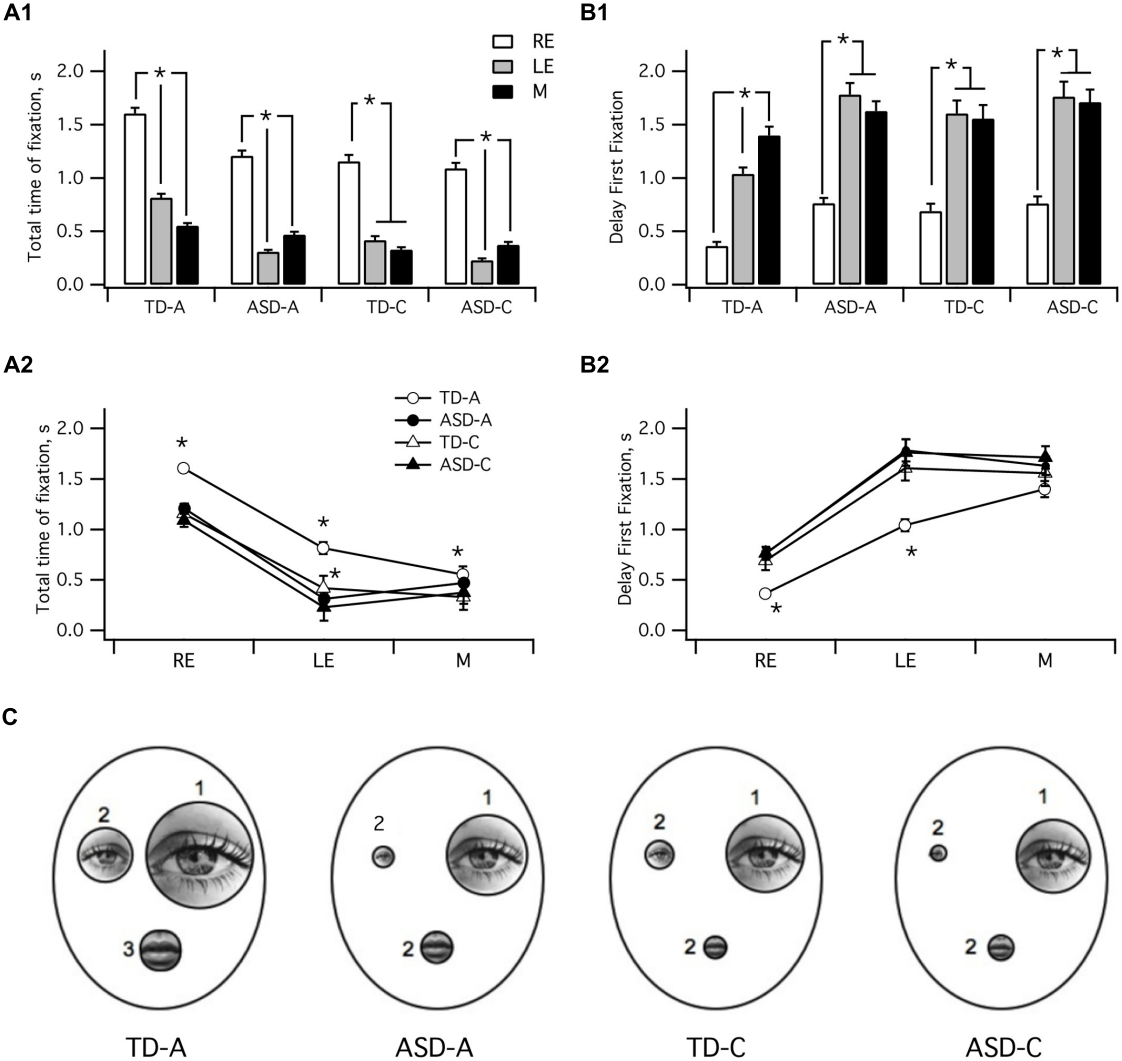
**Characteristics of visual fixations in the four groups. (A)** mean total time spent on fixation: TD-A (LE > RE > M *p* < 0.01); TD-C [LE > (M = RE) *p* < 0.01]; ADL/ASD (LE > M > RE *p* < 0.01); ASD-C (LE > M > RE *p* < 0.01). **(B)** Delay to first fixation in TD-A was shorter than in TD-C or both ADS groups. **(C)** Summary schematic comparing data for the four groups. The size of each area is proportional to the mean total fixation time and the number beside each area indicates the sequence of fixation. TD-A, typical adult; TD-C, typically developing children; ASD-A, adults with ASD; ASD-C, children with ASD. M, mouth; LE, left eye; RE, right eye. ^∗^ indicates a significant variation.

The face exploration strategy was also assessed using the *a posteriori* method of the present study (see **Figures 2** and **3**). The graphs in **Figure 5** present the values for the various parameters analyzed in each ROI. The same data values were either categorized by group to allow direct comparison within groups or by ROIs to allow intergroup comparisons.

As shown in **Figure 5A1**, typical adults spent significantly more time on the RE than either the LE or the mouth (Kruskal–Wallis *H* test; TD-A: RE > LE > M; *H*(2) = 274, *p* < 0.001, *r* = 0.35). Subjects from the TD-C group also spent significantly longer looking at the RE than the mouth or the LE [TD-C : RE > M = LE; *H*(2) = 202, *p* < 0.001, *r* = 0.32], as did both adults with ASD [ASD-A: RE > M > LE; *H*(2) = 308, *p* < 0.001, *r* = 0.25] and children with ASD [ASD-C: RE > M = LE; *H*(2) = 244, *p* < 0.001, *r* = 0.38]. Kruskal–Wallis test and *post hoc* analysis also showed that typical adults spent significantly more time on each ROI than TD-C and ASD subjects [**Figure 5A2**; RE: *H*(3) = 7 3, *p* < 0.001, *r* = 0.06; LE : *H*(3) = 230, *p* < 0.001, *r* = 0.25; M *H*(3) = 47, *p* < 0.001, *r* = 0.04]. TD-C group subjects spent significantly more time on the LE than ASD-C (*p* < 0.001) and ASD-A (*p* = 0.009) but not on the RE (*p* = 1).

To further decipher the face exploration strategy we investigated the temporal pattern of fixation sequences for the various ROIs by considering the delay to the first fixation in each region (**Figure 5B**). Kruskal–Wallis test showed that typical adults scanned the three ROIs sequentially in the following order : RE–LE-M, *H*(2) = 240, *p* < 0.001, *r* = 0.33. Adults with ASD also first looked at the RE, *H*(2) = 308, *p* < 0.001, *r* = 0.25, but they subsequently switched either to mouth or the LE (LE versus M, *p* < 0.055): RE-M/LE. TD-C subjects first looked at the RE *H*(2) = 101, *p* < 0.001, *r* = 0.2, then indifferently the RE or the mouth (LE versus M, *p* = 1): RE–LE/M. Children with ASD also first looked at the RE, *H*(2) = 80, *p* < 0.001, *r* = 0.17, but they then switched arbitrarily to the mouth or the LE RE-LE/M, (LE versus M, *p* = 1).

Kruskal–Wallis test indicated that the scanning strategy presented significant differences between groups for the right and LE [**Figure 5B2**; RE: *H*(68) = 33, *p* < 0.001, *r* = 0.06; LE: *H*(3) = 50, *p* < 0.001, *r* = 0.07] but not for the mouth (*p* = 0.55). *Post hoc* analysis indicated that the RE and LE first fixation delays were significantly shorter in the TD-A group than in the three other groups (**Figure 5B2**, RE: *p* < 0.001; LE: *p* < 0.001). Our data on the face scanning strategies employed by the four groups are summarized in **Figure 5C**. The time spent on each ROI is correlated to its drawn outline size and the number besides each ROI indicates the temporal pattern of first fixation.

## Discussion

### Methodological Considerations

One major pitfall, common to most eye tracking studies is the *a priori* definition of the visual targets (Henderson et al., 2005; Barton et al., 2006; Over et al., 2006). It is only recently that interest has turned to *a posteriori* mapping of visual scenes (Over et al., 2006; Caldara and Miellet, 2011; Falck-Ytter et al., 2013b; Yi et al., 2014) to avoid the subjective definition of ROIs which could potentially explain the absence of consistent findings across studies and compromise the ability to replicate findings (Caldara and Miellet, 2011). In the present study, we have incorporated both spatial normalization to create a prototypic face (Saether et al., 2009) and statistical spatial analysis of fixation distribution to identify significant functional visual targets. The accuracy of our method for identifying significant clusters is demonstrated in **Figure 2C**, where subjects were requested to look at the targets of the calibration map. In this case, it was clearly apparent that only the fixation clusters of relevant interest were retained. Furthermore, to avoid restrictive analysis, the face was subdivided into three large ROIs, each of which included one of the core features previously established as visual targets (Yarbus, 1967). In comparison with the *a priori* method (**Figure 3**), it was clearly evident that this approach provided more significant insights from the same data. To date there is no specific reason for favoring use of a particular method such as that presented here, rather than one based on methodologies used in functional magnetic resonance imaging. In the future, however, it would be relevant to test whether comparable results can be extracted from the same data since, although extremely powerful, the iMap method still requires an experimenter adjusted variable (Gaussian kernel) to characterize the visual information according to the specific hypothesis in question (Caldara and Miellet, 2011).

One issue in developmental studies is to ensure that the reported differences are not due to age specific features such as attention control or task understanding. In the present study, we hypothesized that the use of a free viewing task to measure the spontaneous behavior of our participants would limit the impact of the instructions given to the participants, as for example compared to instructions provided when a specific task (recognition task, gender task…) is achieved.

An analysis of total fixation time (**Table 2**) showed that all groups, with the exception of typical adults, did not exhibit significantly different values, suggesting that there was not a disengagement of general attention for the task in the ASD population during the present free viewing task. This is in agreement with a previous study that found no difference between free-viewing and task-directed conditions (Pelphrey et al., 2002). However, the static stimuli used here, although also suitable for standardizing analysis, are limited in not being as ecological as dynamic stimuli (Klin et al., 2002b; Ponnet et al., 2004).

### Strategies of Visual Fixations

In the present study, spatial statistical analysis has allowed a significant definition of the fixation pattern of human face exploration to be made. Our results confirm the structural importance of inner core features and the presence of a sequential routine of fixation in typical adults. Surprisingly, although the temporal pattern is related to the classical “face information triangle,” i.e., the eyes and mouth, the precise spatial location of these targets does not match the points of interest that are usually reported, i.e., around the pupil, nose, or mouth. In fact, the barycenters of the ROIs established by our *a posteriori* analysis are delocalized downward (**Figure 3**). These results are in accordance with recent findings indicating that in typical adults during a recognition task, the preferred landing positions for the first two fixations is beside the eye rather than being centered on the pupil (Hsiao and Cottrell, 2008; Yi et al., 2014). This infraorbital region (see green dot **Figure 3**) has been suggested to play a crucial role as a center of gravity that from the first glance maximizes the capture of information (Saether et al., 2009). Our results support recent findings by van Belle et al. (2010) since we find that the typical mature pattern of fixations on faces starts at a specific point: below the RE (from the observer’s perspective) before switching to the LE and then to the mouth. In children, a comparable specific region, beyond the eyes, has also been identified using either bubble methods (Spezio et al., 2007b; Wang et al., 2011) or eye tracking in free viewing or in recognition tasks (Hernandez et al., 2009; Yi et al., 2014). The eye avoidance hypothesis (Tanaka and Sung, 2013) provides a plausible explanation of face recognition deficits where individuals with ASD may avoid the eye region because it is perceived as socially threatening. Our results support this hypothesis in both populations (typical and ASD groups): direct eye contact may elicit an automatic avoidance response in humans.

Despite an extensive use of eye tracking in adults in the field of face scanning, this technique has so far been underutilized in research with typical children or adolescents (Karatekin, 2007). To our knowledge, only two developmental studies have examined eye movements during face perception and compared child and adult scanning strategies (Marcus, 2005; Schwarzer et al., 2005). Until now, however, there is no available data that focuses on the pattern of fixation in typical development with static neutral face stimuli in free viewing tasks. As shown in **Figure 5**, the typical adult sequential order of fixation between the three ROIs is neither found in typical children nor in individuals with ASD. Children (typical or with ASD) most frequently start looking at the RE, but subsequently they indifferently look at the LE or the mouth. Since all four sub-groups were tested under the same conditions and methodology, it therefore becomes possible to draw strong conclusions about the specificity of the scan pathway in individuals with ASD compared to typical subjects.

Our results indicate that a different scanpath is employed by TD-A compared to the three other groups. This scanning strategy includes an automatic routine with an alternate visual scan first on the RE then the LE and finally the mouth. In accordance with previous results, therefore, attention is first focused on the eyes, which play a central role in the recognition process and to infer others’ intention (Walker-Smith et al., 1977; Davies et al., 1994; Vuilleumier, 2005; Tanaka and Sung, 2013). By anchoring gaze on this infraorbital region, one perceives the entire face and uses it for face identification (Saether et al., 2009). Our results also provide insights into the development of face processing. The developmental process progressively brings subject behavior toward an optimized strategy in order to capture as quickly as possible the maximum of information. Our observations therefore indicate that while first looking predominantly at the RE, young and ASD subjects indifferently look at the mouth and LE. The observation of such a behavior is in agreement with the results of Barton et al. (2006), who tested the effect of “expertise” by presenting inverted faces that do not access an orientation-dependent face-expert processor, and reported that it elicited a less predictable scan structure.

### Developmental and Pathological Aspects of Face Scanning

Comparing face scanning strategies between the groups indicated that typical children exhibit a distinct immature pattern (relative to typical adults) in which there is no specificity in the temporal sequence of fixation and the length of time spent in fixating the LE or mouth (**Figure 5**). These two features could be therefore considered as indicators of a developmental scanning strategy process, and would in turn be consistent with recent studies proposing that attention to mouth is related to language onset (Hunnius and Geuze, 2004; Young et al., 2009; Nakano et al., 2010).

Regardless of the detailed temporal features of scanning strategy, all four groups studied here paid attention to the same ROIs, and few fixations occurred significantly outside of these areas. Surprisingly, however, we found that both adults and children with ASD adopt a face scanning strategy similar to that of typical children (**Figure 5**, **Table 2**), although the ASD groups differed from their age-matched group regarding the total fixation duration on both eyes (**Figure 5**). Our findings are consistent with other reports that analyzed the eye region by combining fixations for the two eyes (Jones et al., 2008; Hernandez et al., 2009; Bal et al., 2010; Nakano et al., 2010; Yi et al., 2013) but they also extend these previous results by separating the total time spent on each eye and finding that the two ASD groups significantly spent much less time on the LE. The time spent on the RE did not differentiate the two child groups. One possible explanation is that subjects with ASD do not develop an automatic pattern because their atypical processing and/or diminished expertise during childhood do not “drive” visual processing toward a stereotyped pattern as expressed by typical adults. During typical development, humans are socially motivated to be attentive to faces, obliging them to precociously extract relevant information (Senju and Johnson, 2009a,b). In contrast, a social motivation deficit, as encountered in individuals with ASD, may lead to face underexposure and to a disruption in development of the brain systems dedicated to processing faces in a typical pattern (van der Geest et al., 2002; Best et al., 2010). This idea is strengthened by the correlation between social interaction scores and the time spent on the eyes in individuals with autism. However, our results are not in favor of the excess eye/diminished mouth gaze fixation hypothesis proposed in recent studies (Fletcher-Watson et al., 2009; Best et al., 2010; Falkmer et al., 2011), suggesting that the mouth is more likely to be a facial characteristic whose relevance varies according to emotional expression in face exploration, or to movement related to speech (Corden et al., 2008; Hernandez et al., 2009; Norbury et al., 2009; Nakano et al., 2010).

## Conclusion

We propose here a simple method that allows spatial normalization of face stimuli and a statistical data-driven method of extracting eye tracking information. A main strength of the present study is that for the first time, a study based on an *a posteriori* data-based approach was employed for face scanning in a variety of different sub-groups, thereby allowing distinguishing factors that depend on developmental versus pathological processes to be readily deciphered. Based on the present results, the patterns of fixation for static faces that mature from childhood to adulthood in typical subjects are not found in adults with ASD. The atypical patterns found after developmental progression and experience in ASD groups appear to remain blocked in an immature state that cannot be differentiated from typical developmental child patterns of fixation.

## Conflict of Interest Statement

The authors declare that the research was conducted in the absence of any commercial or financial relationships that could be construed as a potential conflict of interest.
